# Field Evaluation of Black PE Ground Cover Against *Rhagoletis batava obscuriosa*: A Two-Year Field Study on a Physical Barrier Technology in Sea Buckthorn Orchards

**DOI:** 10.3390/insects17060613

**Published:** 2026-06-10

**Authors:** Yang Zhou, Adil Sattar, Jipeng Jiao

**Affiliations:** 1 College of Forestry and Landscape Architecture, Xinjiang Agricultural University, Urumqi 830052, China; zy19834545375@163.com (Y.Z.); adl1968@126.com (A.S.); 2 Key Laboratory of Integrated Pest Management on Crops in Northwestern Oasis, Ministry of Agriculture and Rural Affairs, Institute of Plant Protection, Xinjiang Academy of Agricultural Sciences, Urumqi 830091, China; 3 Xinjiang Key Laboratory of Agricultural Biosafety, Institute of Plant Protection, Xinjiang Academy of Agricultural Sciences, Urumqi 830091, China

**Keywords:** *Rhagoletis batava obscuriosa*, physical barrier, black PE ground cover, soil-surface barrier, integrated pest management

## Abstract

To address the “3R” issues (resistance, resurgence, and residue) associated with chemical control of the sea buckthorn fruit fly (*R. batava obscuriosa*), this study proposes a novel physical barrier technology aimed at reducing pesticide application intensity, mitigating environmental pollution, and enhancing fruit quality. Yellow sticky traps were deployed to monitor adult occurrence dynamics and delineate the critical control window, while black polyethylene (PE) ground cover was installed on the orchard floor around the base of sea buckthorn trunks to prevent adult emergence from the soil. Control efficacy was evaluated by comparing adult trap catches and fruit infestation rates between the black PE ground cover treatment and the untreated control. Monitoring results revealed that adult emergence commenced on 29 June, entered the peak period on 9 July, attained maximum trap catch on 24 July, and persisted into the late emergence phase through mid-to-late August. Control data demonstrated that mean trap catches in the black PE ground cover treatment were lower than those in the control. From 2024 to 2025, fruit infestation rates declined from 74.5% and 62.3% in the control plot to 19.0~22.0% and 16.2~19.3% in the treatment plots, respectively, with control efficacy consistently exceeding 65%. This study demonstrates that black PE ground cover reduces adult abundance and fruit infestation rates of *R. batava obscuriosa*, with control efficacy consistently exceeding 65%. The observed effects are consistent with a soil-surface barrier effect and likely attributed to dual physical mechanisms: it may reduce adult emergence from the soil into the canopy and may obstruct mature larvae from entering the soil to pupate. This technology represents an environmentally sound, sustainable green control option suitable for integration into IPM programs for the sea buckthorn industry.

## Simple Summary

In Qinghe County, Altay, the sea buckthorn fruit fly (*Rhagoletis batava obscuriosa*) is univoltine, completing one generation annually and overwintering as pupae in the soil. Adult emergence spanned approximately 55 days, from 29 June to mid-to-late August, with the peak emergence period commencing on 9 July and maximum trap captures recorded on 24 July. Following the deployment of black polyethylene (PE) ground cover on the orchard floor, fruit infestation rates decreased markedly from 74.5% (2024) and 62.3% (2025) in the control plot to 19.0~22.0% and 16.2~19.3% in the treatment plots, respectively. Control efficacy remained consistently above 65% across both years. These results suggest that this technology likely achieves environmentally sound, green pest management through two plausible physical mechanisms: it may reduce adult emergence from the soil into the canopy and may obstruct mature larvae from entering the soil to pupate.

## 1. Introduction

Sea buckthorn (*Hippophae rhamnoides* L.), a deciduous shrub or small tree belonging to the genus *Hippophae* within the family Elaeagnaceae, is widely distributed across Eurasia [1,2]. China possesses the largest sea buckthorn forest area in the world, totaling approximately 2.26 million hectares and accounting for 88% of the global sea buckthorn forest area [3,4]. Xinjiang represents a key distribution region for sea buckthorn resources in China, with Altay Prefecture accounting for the largest cultivation area. Large-scale sea buckthorn plantations have been established in Qinghe County, Burqin County, and Habahe County, among other localities.

The sea buckthorn fruit fly (*Rhagoletis batava obscuriosa*) belongs to the genus *Rhagoletis* (Diptera: Tephritidae). It is host-specific, feeding exclusively on sea buckthorn fruits. Adult females oviposit beneath the fruit epidermis; upon hatching, the larvae bore into the pulp and feed internally. Infested fruits subsequently shrivel and desiccate, often leaving only the dried peel, thereby severely impairing fruit quality and marketability [5]. This pest is primarily distributed across northern European countries, including Russia, Germany, and Belarus [6,7]. In China, *R. batava obscuriosa* was first detected in Jianping County, Liaoning Province, in 1985, and has subsequently been reported in Shanxi, Shaanxi, Heilongjiang, Qinghai, and other provinces [8]. The life history characteristics of the *R. batava obscuriosa* present significant challenges for management. It is univoltine, completing one generation per year and overwintering primarily as pupae in the soil. The timing of adult emergence and the duration of the occurrence period vary slightly among geographic regions [9,10,11,12]. During outbreaks, fruit infestation rates often exceed 80% and may reach 100% under severe infestation conditions [13].

Physical control refers to a technical approach that utilizes insect tropisms (e.g., color preference, chemical attraction) or artificially intervenes in their living environment to reduce pest population density. It is characterized by being environmentally friendly and residue-free, and constitutes an important component of integrated pest management (IPM) systems for tephritid fruit flies. Current physical control methods for tephritid fruit flies mainly include colored sticky traps, attractant application, fruit bagging, and soil turning to eliminate pupae [14].

Regarding color-based trapping, exploiting the color preference behavior of fruit flies for monitoring and control has been widely applied. For instance, yellow sticky traps exhibit high capture efficacy against *Bactrocera dorsalis*, *Carpomya vesuviana*, and *R. batava obscuriosa*. Regarding the application of attractants, research on *R. batava obscuriosa* has demonstrated that combining a slow-release attractant with yellow sticky traps achieves greater trapping efficacy than using homemade traps [15]. Studies on the European sea buckthorn fruit fly (*Rhagoletis batava*) have confirmed that specialized yellow sticky traps increase trap catches by 30~50% compared to standard yellow sticky traps, and a synergistic trapping technique integrating colored sticky cards with attractants has been proposed [16].

However, existing physical control techniques still face challenges in practical application, including limited trapping and killing efficacy and susceptibility to meteorological disturbances. Therefore, exploring novel physical barrier technologies based on ground mulching is of considerable significance for addressing the shortcomings of current methods in pest management.

In recent years, severe outbreaks of *R. batava obscuriosa* have occurred in Xinjiang. Since 2014, this pest has infested large areas of the Altay Prefecture, and its presence has been successively detected in Urumqi County, Akqi County, and Qinghe County, causing substantial economic losses to the local sea buckthorn industry and severely constraining its development [17]. Current control measures for *R. batava obscuriosa* primarily target the adult stage; however, the adult emergence period is protracted and asynchronous. Therefore, accurately elucidating adult occurrence dynamics is fundamental for identifying the critical control window and enhancing management efficiency.

To this end, a systematic monitoring program of adult occurrence dynamics of *R. batava obscuriosa* was conducted in Qinghe County in 2024. Adult population fluctuations were recorded, and occurrence dynamics curves were constructed to delineate the critical temporal window for control interventions. Based on the aforementioned biological characteristics—specifically, the behavior of mature larvae pupating in the soil primarily within a 60 cm radius of the sea buckthorn trunk base [17]—this study deployed black PE ground cover on the ground surrounding the trunks to establish a physical barrier. This study systematically evaluated the field control efficacy of black PE ground cover against *R. batava obscuriosa* by comparing adult abundance and fruit infestation rates between treatment and control plots over two consecutive years. We analyzed its potential role in disrupting the life cycle of *R. batava obscuriosa* by potentially interfering with adult emergence and larval pupation, explored the advantages and limitations of this technology, and preliminarily assessed its practical application prospects in sea buckthorn orchards. This study aims to provide a scientific basis for establishing green and sustainable integrated pest management strategies against *R. batava obscuriosa*.

## 2. Materials and Methods

### 2.1. Experimental Site Description

The experimental site was located in Wobate Township, Qinghe County, Altay Prefecture, Xinjiang Uygur Autonomous Region, China (46°33′51″ N, 90°15′35″ E). The primary sea buckthorn cultivars planted in the area were ‘Zhuangyuanhuang’ and ‘Shenqiuhong’. The orchard floor was densely covered with weeds, and management practices were extensive, consisting solely of winter pruning and flood irrigation. Natural infestation by *R. batava obscuriosa* was severe at the site. The adult occurrence dynamics monitoring experiment and the black PE ground cover barrier experiment were conducted in independent adjacent plots within the same orchard, and all experimental plots were separated by a distance of 50 m to prevent mutual interference between treatments.

### 2.2. Experimental Materials

The following materials were utilized in this study: label tags; yellow sticky traps (Beijing Zhongjie Sifang Biotechnology Co., Ltd., Beijing, China; wavelength 570 nm, PVC board, 25 cm × 25 cm); black PE ground cover (Ningbo Yingmi Trading Co., Ltd., Ningbo, China; 1.2 m × 1.2 m, 0.1 mm thickness); and black PE ground cover fixing pegs. The black PE ground cover is manufactured using a weaving process with extruded plastic flat filaments. The black PE ground cover is formulated with UV stabilizers to enhance outdoor durability and exhibits excellent abrasion resistance, compression resistance, and chemical corrosion resistance. The black PE ground cover has good water permeability. This water permeability characteristic of the black PE ground cover allows irrigation water to infiltrate while effectively reducing soil water evaporation, providing good water and moisture conservation effects.

### 2.3. Monitoring of Adult Occurrence Dynamics of R. batava obscuriosa

Monitoring of adult population dynamics of *R. batava obscuriosa* was conducted from June to September 2024. Three sample plots with comparable sea buckthorn tree age and site conditions were selected as replicates, each covering an area of 1600 m^2^. Yellow sticky traps were suspended within the orchard to monitor adult population dynamics. In each plot, five yellow sticky traps were deployed following a diagonal sampling pattern. Traps were hung on the northern side of the canopy, adjacent to the main trunk, at a height of 1.5 m above ground level, in locations with abundant fruit clusters. The number of adult *R. batava obscuriosa* captured was recorded, and the sticky traps were replaced every 5 days. Based on the monitoring data, an adult occurrence dynamics curve was constructed to determine the critical temporal window for control interventions. Five yellow sticky traps were deployed per plot in this monitoring experiment.

### 2.4. Black PE Ground Cover Barrier Experiment

#### 2.4.1. Deployment of Black PE Ground Cover

**Figure 1:**
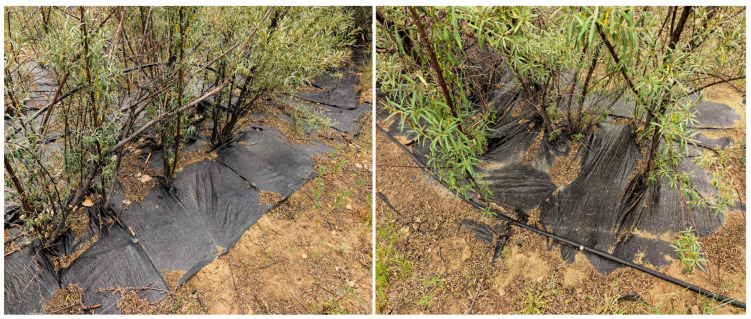
Representative photographs of the experimental setup are shown.

**Figure 2:**
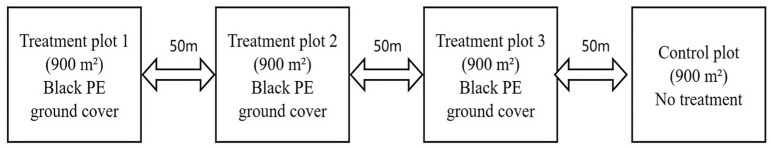
Schematic diagram of the black PE ground cover experimental treatments.

The experiment was conducted over two consecutive years, 2024 and 2025, in Qinghe County. Black PE ground cover was installed prior to the emergence of adult *R. batava obscuriosa* from the soil (Figure 1). Four sample plots with comparable tree age and site conditions were selected to ensure that the experimental area was representative and could reliably reflect the control efficacy of the black PE ground cover against *R. batava obscuriosa*. Each plot covered an area of 900 m^2^, and plots were separated by a distance of 50 m (Figure 2). Three plots were designated as treatment plots, in which black PE ground cover was deployed, and one plot served as the untreated control without fabric coverage. Within each treatment plot, black PE ground cover was extensively laid on the ground surface within a 60 cm radius centered on the trunk of each sea buckthorn tree. This radius was selected based on the quantitative survey results of Zhang [17], who investigated the pupal distribution of *R. batava obscuriosa* in Altay Prefecture. The study systematically counted the number of pupae in soil samples collected from different radius ranges around 20 sea buckthorn trees, and the results showed that 91.86% of pupae were distributed within a 60 cm radius of the trunk base. Specifically, 46.91% of pupae were found in the 0–20 cm radius range, 24.91% in the 21–40 cm radius range, 20.04% in the 41–60 cm radius range, and only 8.14% beyond 60 cm. The perimeter edges of the fabric were covered with soil and firmly compacted to ensure a flat, seamless barrier that prevented the escape of adult *R. batava obscuriosa*. The integrity of the fabric was inspected periodically throughout the experimental period, and any detected damage was promptly repaired. The control plot received no such intervention. It should be noted that the control treatment was not replicated at the plot level in this study. The three yellow sticky traps and ten sample trees within the control plot were treated as subsamples rather than independent replicates.

#### 2.4.2. Adult Population Monitoring in Treatment Plots

In each plot, three yellow sticky traps were deployed following a diagonal sampling pattern and suspended at a height of 1.5 m above ground level to continuously monitor adult abundance and activity. Trap catches were recorded and sticky traps were replaced every 5 days. Adult counts of *R. batava obscuriosa* were documented in detail. Trap catch data were collected from 4 July to 8 August, encompassing a total of eight survey occasions. This monitoring period was selected because it covered the entire effective damage period of *R. batava obscuriosa*. Adults were first captured on 29 June 2024, but cumulative catches from 29 June to 4 July accounted for only 2.5% of the total annual catch, with low impact on fruit damage. By 8 August, cumulative catches had reached 93.6% of the total annual catch. Total trap catch for each treatment was calculated as the cumulative number of adults captured across all three yellow sticky traps over the eight survey dates. Maximum trap catch was defined as the highest single-trap capture recorded during any single survey occasion within this period. Mean trap catch per treatment was expressed as the average ± standard deviation of the three yellow sticky traps across the monitoring period.

#### 2.4.3. Assessment of Fruit Infestation Rate

Larvae of *R. batava obscuriosa* feed internally on sea buckthorn fruits, which do not abscise prematurely; desiccated, damaged fruits often remain attached to branches and are clearly visible in the following year. Larval exit holes or oviposition punctures are readily discernible on the fruit epidermis. Based on adult population occurrence dynamics monitoring, fruit infestation rates were assessed annually on 20 July in both treatment and control plots. This date was selected based on two key biological characteristics of *R. batava obscuriosa*: adults require a 3–5 day pre-oviposition period for nutritional supplementation after emergence, and the oviposition peak typically occurs 3–5 days earlier than the adult trap catch peak. According to the 2024 monitoring data, the adult trap catch peak occurred on 24 July, so the oviposition peak was estimated to be 19–21 July. Within each plot, five sampling points were established following a five-point sampling pattern. At each sampling point, two sea buckthorn trees with more than half of the fruits exhibiting color change were selected as sample trees, yielding a total of ten sample trees per plot. On each sample tree, one standard branch was selected and labeled, and 100 fruits on that branch were examined for infestation. Infested fruits were identified by the presence of larval exit holes or oviposition punctures visible on the fruit epidermis, and the number of infested fruits was recorded.

### 2.5. Data Analysis

Adult occurrence dynamics monitoring data and trap catch data of *R. batava obscuriosa* from the experimental plots were compiled using Microsoft Excel 2019. Graphics were generated using Origin 2024. Three yellow sticky traps were deployed per plot in the black PE ground cover barrier experiment. The 10 sample trees and 3 traps within the single control plot were treated as subsamples, not true independent replicates.

Descriptive statistics (mean ± standard deviation) are used to present the control plot data as a biological baseline reference. The magnitude of the control effect was evaluated by calculating the percentage reduction in adult trap catches and fruit infestation rates between treatment and control plots.

### 2.6. Calculation of Fruit Infestation Rate and Control Efficacy

The fruit infestation rate and control efficacy were calculated using the following formulas:

(1) $$\mathrm{Fruit}\;\mathrm{infestation}\;\mathrm{rate}=\frac{\mathrm{Number}\;\mathrm{of}\;\mathrm{infested}\;\mathrm{fruits}\;\mathrm{surveyed}}{\mathrm{Total}\;\mathrm{number}\;\mathrm{of}\;\mathrm{fruits}\;\mathrm{surveyed}}\;\times \;100\%$$

(2) $$\mathrm{Control}\;\mathrm{efficacy}\;(\%)=\frac{\mathrm{Control}\;\mathrm{infestation}\;\mathrm{rate}\;-\;\mathrm{Treatment}\;\mathrm{infestation}\;\mathrm{rate}}{\mathrm{Control}\;\mathrm{infestation}\;\mathrm{rate}}\;\times \;100\%$$

## 3. Results

### 3.1. Adult Occurrence Dynamics of R. batava obscuriosa in Qinghe County

**Figure 3:**
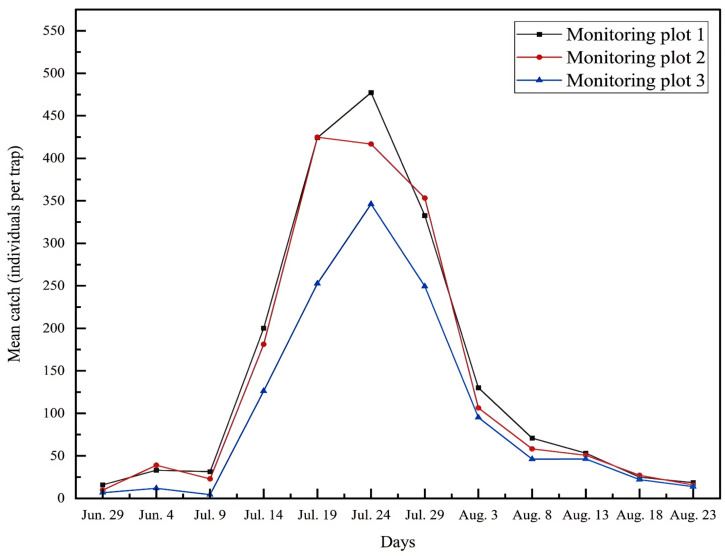
Adult occurrence dynamics of *R. batava obscuriosa* in Qinghe County.

Monitoring surveys of *R. batava obscuriosa* commenced in June 2024. The results revealed that the entire adult emergence period lasted approximately 55 days, with the population dynamics of adult *R. batava obscuriosa* illustrated in Figure 3. Adults were first captured in yellow sticky traps on 29 June, indicating the onset of the emergence period and a subsequent upward trend in population abundance; i.e., adult emergence from the soil commenced in late June. The period from 29 June to 9 July corresponded to the initial emergence phase. Beginning on 9 July, the number of emerging adults increased sharply, signifying entry into the peak emergence period. Adult emergence was relatively concentrated during this phase, during which population density attained its maximum. The highest single-day total trap catch was recorded on 24 July, with Plot 1 yielding a single-day catch of 2387 adults. The peak period extended through late July, after which the number of emerging adults declined markedly, indicating the late emergence phase, which persisted until mid-to-late August.

### 3.2. Control Efficacy in the Black PE Ground Cover Experimental Plots in 2024

As presented in Table 1, the black PE ground cover treatment plots showed lower trap catches and fruit infestation rates compared with the untreated control plot in 2024. The trap catches across the three treatment plots were broadly consistent, suggesting stable performance of the ground cover under the conditions of this field trial. However, because the untreated control was not replicated at the plot level, these results should be interpreted descriptively rather than as formal statistical evidence of reproducibility. In terms of total trap catch, the control recorded the highest cumulative capture, with 4139 adults trapped during the monitoring period. The maximum trap catch in the control was 430 adults per trap per 5-day interval, and the mean catch was 517.38 ± 161.75 individuals per 5-day interval. In contrast, the maximum trap catch in the treatment plots was 205 individuals per trap per 5-day interval.

**Figure 4:**
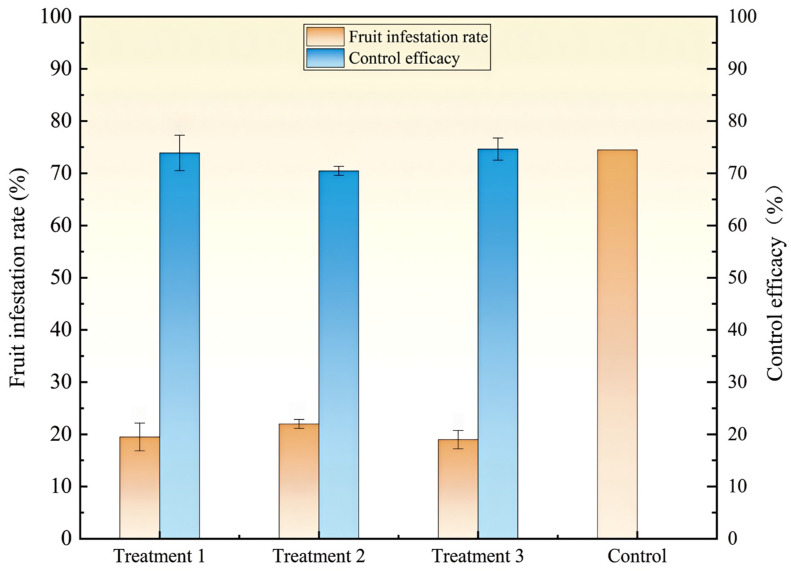
Effects of black PE ground cover treatment on fruit infestation rate and control efficacy against *R. batava obscuriosa* in 2024. Note: Error bars represent standard deviation (SD) of the mean values from ten sample trees per plot.

The results of the fruit infestation survey (Figure 4) revealed that infestation rates in the treatment plots ranged from 19% to 22%, which were lower than the 74.5% recorded in the control plot. Control efficacy in all treatment plots exceeded 70%, indicating that the deployment of black PE ground cover on the orchard floor provides effective control of *R. batava obscuriosa* and represents a viable, environmentally sound management strategy for this pest.

### 3.3. Control Efficacy in the Black PE Ground Cover Experimental Plots in 2025

As presented in Table 2, the black PE ground cover treatment plots showed lower trap catches and fruit infestation rates compared with the untreated control plot in 2025. The trap catches across the three treatment plots were broadly consistent, suggesting stable performance of the ground cover under the conditions of this field trial. However, because the untreated control was not replicated at the plot level, these results should be interpreted descriptively rather than as formal statistical evidence of reproducibility. Regarding total trap catch, the control recorded the highest cumulative capture of *R. batava obscuriosa* during the monitoring period, totaling 878 adults. The mean catch was 109.75 ± 20.23 individuals per 5-day interval, and the maximum catch was 122 individuals per trap per 5-day interval.

**Figure 5:**
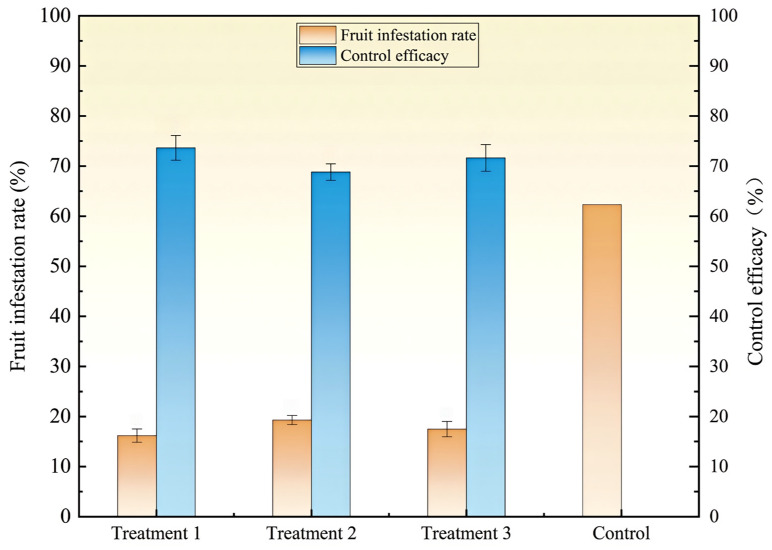
Effects of black PE ground cover treatment on fruit infestation rate and control efficacy against *R. batava obscuriosa* in 2025. Note: Error bars represent standard deviation (SD) of the mean values from ten sample trees per plot.

A fruit infestation survey was conducted in the black PE ground cover treatment area (Figure 5). In 2025, the fruit infestation rate in the control plot was 62.3% ± 1.88%, whereas infestation rates in the treatment plots ranged from 16.2% to 19.3%. Control efficacy values ranged from 68.81% to 73.65%. These results further demonstrate that the deployment of black PE ground cover on the orchard floor provides substantial control efficacy against *R. batava obscuriosa*.

## 4. Discussion

Systematic monitoring of pest population occurrence dynamics to elucidate their spatiotemporal patterns constitutes the foundation for determining critical control periods and formulating scientifically sound management strategies. In this study, yellow sticky trap monitoring was employed to delineate the adult population occurrence dynamics of *R. batava obscuriosa* in Qinghe County, Altay Prefecture, Xinjiang. Consistent with previous reports on the biology of *R. batava obscuriosa* [17], this species is univoltine in the Altay region, overwintering as pupae in the soil, and adults emerge from mid-June to early August each year.

The results of this two-year field study demonstrate that black PE ground cover provides effective and sustained control of *R. batava obscuriosa* in sea buckthorn orchards. This soil-surface physical barrier targets the unique soil-pupating biology of *R. batava obscuriosa*, and may disrupt two critical stages of its life cycle by potentially reducing adult emergence from overwintering pupae and obstructing mature larvae from entering the soil to pupate. Notably, the total adult trap catch in the control plot was 4139 individuals in 2024, but only 878 individuals in 2025. This substantial interannual variation should be interpreted cautiously, as it is likely driven by a combination of natural and external factors rather than the experimental treatment alone. First, natural population variability and climatic conditions can strongly influence the population dynamics of this univoltine pest. Differences in winter soil temperatures, moisture, or extreme weather events between the two years may have significantly impacted the overwintering survival rates of pupae. Second, external area-wide pest control activities acted as a major confounding factor; during the occurrence period, the local government organized drone aerial spraying in the areas surrounding the experimental orchard, which likely contributed to the overall population decline in 2025. Finally, while the black PE ground cover deployed in 2024 locally blocked mature larvae from entering the soil, its suppressive effect was not directly tested at the whole-orchard scale and should not be overemphasized.

The lower adult abundance observed in 2025 should be interpreted cautiously because it may reflect both the possible suppressive effect of the black PE ground cover and external area-wide pest control activities, including government-organized drone spraying near the experimental orchard.

Importantly, this interannual variation does not compromise the validity of the within-year control efficacy estimates, as both the treatment and control plots were equally exposed to the same external conditions during each experimental year.

*R. batava obscuriosa* overwinters as pupae in the surface layer of the soil, and mature larvae exhibit the behavior of exiting the fruit and dropping to the ground to pupate. Female adults deposit their eggs beneath the epidermis of the fruit [15]. Based on the well-documented soil-pupating biology of *R. batava obscuriosa* and the reductions in adult trap catches and fruit infestation observed in treatment plots, two plausible physical mechanisms are proposed to explain the pest suppression effect. First, the black PE fabric deployed on the orchard floor may act as a physical barrier that reduces adult emergence from the soil into the canopy, thereby potentially inhibiting oviposition behavior. Second, the ground cover may simultaneously obstruct the pathway for mature larvae to enter the soil for pupation, which could effectively reduce the overwintering population base in subsequent generations. This physical exclusion mechanism has been systematically validated in the management of various tephritid and drosophilid fruit fly pests. For drosophilid pests, existing research has primarily focused on the spotted-wing drosophila (*Drosophila suzukii*), a major pest of berry crops. Pioneering work in raspberry orchards employed a combined strategy of high-tunnel insect-proof netting and ground-level plastic film to establish exclusion barriers both aerially and at the soil surface. The netting prevented adult *D. suzukii* from entering the planting area, while the plastic film obstructed mature larvae from dropping to the soil for pupation. Results from this investigation demonstrated that insect-proof netting alone reduced larval infestation rates from 81% to 35%, whereas the combined exclusion technique achieved a control efficacy of 98%, substantially outperforming conventional chemical control [18]. In a subsequent study in raspberry, the application of plastic mulches on the orchard floor simultaneously blocked adult emergence from the soil and larval entry for pupation, while also modifying canopy microclimate conditions. This approach reduced adult *D. suzukii* populations by 42~51% and larval populations by 52~72% [19].

For tephritid fruit fly pests, existing studies have encompassed multiple species, including *B. dorsalis*, *Bactrocera cucurbitae*, and *R. batava obscuriosa*. Precise laboratory bioassays evaluating the barrier efficacy of different materials have demonstrated that ground covers of non-woven fabric and polyethylene (PE) plastic film both completely prevent the penetration of third-instar mature larvae of *B. dorsalis*, with no larvae successfully crossing the barrier after 24 h of exposure [20]. In a field experiment conducted in cucumber orchards, the deployment of black plastic film as a ground cover obstructed both adult emergence from the soil and larval entry into the soil for pupation of *B. cucurbitae*, resulting in a significant reduction in fruit infestation rate from 23.09% to 3.95% [21]. In the present study, the sustained control efficacy observed across two consecutive years is consistent with the hypothesis that the 0.1 mm thick black PE ground cover may obstruct mature larvae of *R. batava obscuriosa* from entering the soil to pupate, thereby reducing the overwintering population base at its source. This is supported by the fact that adult trap catches in treatment plots were lower than those in control plot in both 2024 and 2025, which likely contributes to reduced adult abundance and fruit infestation.

Furthermore, the efficacy of physical exclusion techniques has been corroborated across a broader range of pest taxa. Previous studies have established that the effectiveness of physical exclusion barriers depends on matching mesh size to the body dimensions of the target pest. Ebert demonstrated that screens with rectangular openings must limit the short side to less than the minimum body width of the pest to prevent penetration [22]. In a field trial conducted in citrus orchards in Guangxi, the erection of insect-proof netting walls enclosing the entire orchard to obstruct adult immigration achieved over 92.04% control efficacy against *D. citri* nymphs and over 87.06% efficacy against both adults and eggs, leading to a substantial reduction in chemical pesticide inputs [23].

The findings of the aforementioned studies are mutually corroborative with the results of the present investigation, collectively demonstrating that physical exclusion technologies—exemplified by ground mulching and net-enclosure isolation—possess broad applicability and high efficacy in the management of agricultural and forestry pests. Compared with techniques such as yellow sticky trap monitoring and sex pheromone lures, which require periodic replacement and maintenance, black PE ground cover offers a distinct advantage in terms of long-term durability: a single installation can provide sustained control efficacy for two to three years. This durability characteristic aligns closely with the three-to-five-year service life of insect-proof netting documented in previous research [18], further underscoring the economic feasibility of physical exclusion technologies in long-term pest management programs.

Several methodological limitations of this study should be acknowledged. First, the control treatment was not replicated at the plot level, with only one untreated control plot established. This prevents formal inferential statistical comparisons between treatment and control groups, and the control data are therefore presented as a biological baseline rather than a statistically replicated reference. Second, although plot-level variability in soil properties, vegetation coverage, and pest population density may exist, the descriptive results demonstrated consistent control efficacy among the three treatment replicates, suggesting consistent spatial performance under the conditions of this trial. However, because the untreated control was not replicated at the plot level, these results should be interpreted descriptively rather than as formal evidence of reproducibility. Third, adult *R. batava obscuriosa* have certain flight ability, and possible immigration of adults from the control plot or surrounding unmanaged areas into the treatment plots may have led to an underestimation of the true control efficacy of the black PE ground cover. Fourth, cross-year comparisons of absolute adult abundance are confounded by external area-wide pest control activities conducted in 2025, which may have contributed to the overall population decline observed across all plots. Fifth, this study only conducted two consecutive years of monitoring, which is insufficient to disentangle the relative contributions of natural population variability, climatic conditions, and treatment effects to the observed interannual population changes. Long-term multi-year monitoring across multiple sites is required to quantify these effects. Finally, this study did not conduct direct measurements of larval pupation success or adult emergence rates beneath the ground cover.

In addition, this study has a limitation in mechanism validation: we did not directly quantify larval movement into the soil, pupal survival rates beneath the fabric, or adult emergence success from covered soil. The proposed dual mechanisms are therefore plausible inferences rather than experimentally confirmed conclusions. Future studies should conduct targeted experiments, such as placing marked larvae on the fabric surface to monitor their penetration ability, and installing emergence traps beneath the fabric to directly measure adult emergence rates, to further validate the underlying mechanisms.

From an ecological economics perspective, black PE ground cover technology may offer notable potential economic and environmental advantages compared with repeated chemical applications, aligning with the principles of green agriculture. Compared with conventional chemical control, this approach may eliminate pesticide residues on fruit, thereby potentially preserving fruit quality, potentially reducing negative environmental impacts, and potentially maintaining the population stability of natural enemy insects. Moreover, the fabric may be recyclable and may leave no persistent environmental pollutants upon disposal, conferring potential ecological benefits.

Black PE ground cover may offer economic and environmental advantages compared with repeated chemical applications; however, these potential benefits have not been directly quantified in the present study. Detailed cost–benefit analysis, long-term assessment of material durability under field conditions, and comprehensive evaluation of plastic waste management and environmental fate are needed before large-scale recommendation.

From a practical production perspective, the application of black PE ground cover technology also faces several constraints that need to be addressed. In terms of labor and cost, the installation of ground cover is relatively simple in flat, intensively managed orchards. However, in sloped orchards or extensively managed orchards with dense thorns on sea buckthorn trunks, the installation difficulty increases significantly.

Regarding durability, the 0.1 mm thick black PE ground cover used in this study maintained structural integrity throughout the two-year experimental period, and is expected to remain functional for 2–3 years under normal orchard conditions based on material specifications. However, it is susceptible to tearing from mechanical operations, animal activities, and strong winds. Regular inspections and repairs are required when damage occurs to ensure barrier effectiveness.

For orchard management practices, black PE ground cover has minimal impact on drip irrigation systems. Notably, the ground cover also exhibits a weed suppression effect observed during the experiment, which may partially offset its installation cost. Future research should further explore the synergistic mechanisms between black PE ground cover and other environmentally sound control tactics, with the goal of establishing a multi-tactic integrated pest management framework for *R. batava obscuriosa*.

## 5. Conclusions

This two-year field study indicates that black PE ground cover has strong potential as a soil-surface physical barrier for reducing adult abundance and fruit infestation of *R. batava obscuriosa* in sea buckthorn orchards. However, further validation across multiple orchards, years, topographic conditions, and fully replicated control designs is required before broad-scale recommendation.

Although black PE ground cover shows potential to reduce pesticide application intensity and improve fruit quality and safety in sea buckthorn orchards, these outcomes were not directly assessed in the present study and should therefore be interpreted with caution. If validated by further research, the wider adoption of this technology could contribute to the green and high-quality development of the sea buckthorn industry in the Altay region and areas with similar ecological and agronomic conditions, and may also provide a useful reference for the physical control of other soil-pupating tephritid fruit fly pests.

Future research should focus on three priority directions: (1) optimizing material formulations to enhance outdoor durability and develop biodegradable alternatives to reduce plastic waste; (2) evaluating the adaptability of this technology under different topographic conditions, cultivation modes and climate zones; and (3) exploring the synergistic application of this physical barrier technology with other green control measures to build a more comprehensive integrated pest management system for *R. batava obscuriosa*.

## Figures and Tables

**Table 1:** Trap capture efficacy of black PE ground cover treatment against *R. batava obscuriosa* in 2024.

Treatment	Total Trapped (Individuals)	Highest Trapped (Individuals/5d·Trap)	Mean Trapped (Individuals/5d·Plot)
1	1532	154	191.5 ± 63.95
2	1613	179	201.63 ± 65.60
3	1613	205	201.13 ± 66.70
Control	4139	430	517.38 ± 161.75

Note: Treatments 1–3 were covered with black PE ground cover (three replicate plots). Control was without coverage. Values presented in the tables are mean ± standard deviation of the total trap catch per 5-day interval within each plot. The control plot is presented as a biological baseline only.

**Table 2:** Trap capture efficacy of black PE ground cover treatment against *R. batava obscuriosa* in 2025.

Treatment	Total Trapped (Individuals)	Highest Trapped (Individuals/5d·Trap)	Mean Trapped (Individuals/5d·Plot)
1	164	21	20.5 ± 5.02
2	182	20	22.75 ± 4.53
3	190	27	23.75 ± 5.74
Control	878	122	109.75 ± 20.23

Note: Treatments 1–3 were covered with black PE ground cover (three replicate plots). Control was without coverage. Values presented in the tables are mean ± standard deviation of the total trap catch per 5-day interval within each plot. The control plot is presented as a biological baseline only.

## Data Availability

The original contributions presented in this study are included in the article. Further inquiries can be directed to the corresponding author.
